# Overexpression of CX3CR1 in Adipose-Derived Stem Cells Promotes Cell Migration and Functional Recovery After Experimental Intracerebral Hemorrhage

**DOI:** 10.3389/fnins.2019.00462

**Published:** 2019-05-08

**Authors:** Gaigai Li, Haihan Yu, Na Liu, Ping Zhang, Yingxin Tang, Yang Hu, Ye Zhang, Chao Pan, Hong Deng, Jiahui Wang, Qi Li, Zhouping Tang

**Affiliations:** ^1^Department of Neurology, Tongji Hospital, Tongji Medical College, Huazhong University of Science and Technology, Wuhan, China; ^2^Department of Neurology, The First Affiliated Hospital of Chongqing Medical University, Chongqing, China

**Keywords:** intracerebral hemorrhage, adipose-derived stem cells, migration, fractalkine, FKN, CX3CR1

## Abstract

Stem cell therapy has emerged as a new promising therapeutic strategy for intracerebral hemorrhage (ICH). However, the efficiency of stem cell therapy is partially limited by low retention and engraftment of the delivered cells. Therefore, it’s necessary to improve the migration ability of stem cells to the injured area in order to save the costs and duration of cell preparation. This study aimed to investigate whether overexpression of CX3CR1, the specific receptor of chemokine fractalkine (FKN), in adipose-derived stem cells (ADSCs) can stimulate the cell migration to the injured area in the brain, improve functional recovery and protect against cell death following experimental ICH. ADSCs were isolated from subcutaneous adipose tissues of rats. ICH was induced by means of an injection of collagenase type VII. ELISA showed that the expression levels of fractalkine/FKN were increased at early time points, with a peak at day 3 after ICH. And it was found that different passages of ADSCs could express the chemokine receptor CX3CR1. Besides, the chemotactic movements of ADSCs toward fractalkine have been verified by transwell migration assay. ADSCs overexpressing CX3CR1 were established through lentivirus transfection. We found that after overexpression of CX3CR1 receptor, the migration ability of ADSCs was increased both *in vitro* and *in vivo*. In addition, reduced cell death and improved sensory and motor functions were seen in the mice ICH model. Thus, ADSCs overexpression CX3CR1 might be taken as a promising therapeutic strategy for the treatment of ICH.

## Introduction

Intracerebral hemorrhage (ICH) is a severe neurological disease with high morbidity and mortality (Balami and Buchan, 2012; Rabinstein, 2017). Current treatment options for ICH mainly include surgical evacuation of hematoma, reduction of intracranial pressure, prevention of cerebral edema, and general supportive measures (Mayer and Rincon, 2005; Rabinstein, 2017). In recent years, stem cell therapy has emerged as a new promising therapeutic strategy for ICH (Sun et al., 2015; Hu et al., 2016; Bedini et al., 2018). It is reported that stem cells can promote functional recovery through the mechanisms of directly transdifferentiation, paracrine effects, and immune regulation (Bronckaers et al., 2014; Liu et al., 2014; Wei et al., 2017). Based on the results of these animal studies, clinical trials of stem cells for recent ICH and cerebral hemorrhage sequela are ongoing now (NCT03371329; NCT02283879). Adipose tissue is one of the most abundant and easily accessible sources for mesenchymal stem cell (MSCs), with the advantages of high yield, low immunogenicity and avoidance of ethical issues (Schäffler and Büchler, 2007; Forcales, 2015; Gutiérrez-Fernández et al., 2015). A large number of preclinical studies and several clinical trials have shown that adipose-derived stem cells (ADSCs) transplantation could reduce neuronal apoptosis, promote regeneration of neurons and vessels and improve functional recovery after brain injury (Gimble et al., 2012; Neirinckx et al., 2013; Gutiérrez-Fernández et al., 2015; Konala et al., 2016). It has been found in some studies that ADSCs can improve ICH-induced neurological deficits (Chen et al., 2012; Yang et al., 2012; Kuramoto et al., 2019).

It is generally believed that the efficiency of stem cell therapy to attenuate inflammation, or injuries after ischemia depends on the number of MSCs migrating and homing to the target tissues (Chavakis et al., 2008; Karp and Leng, 2009). Despite the tendency of MSCs migrating to the lesion site, however, only a small percentage of the infused MSCs reach the target tissue, due to the effects of entrapment in peripheral filter organs and the existence of blood-brain barrier (BBB) (Detante et al., 2009; Arbab et al., 2012; Liu et al., 2013; Tang et al., 2015). Furthermore, the exact mechanisms underlying the migration and homing of MSCs are not fully understood. It is proposed that like leukocyte homing, recruitment and incorporation of MSCs into injured tissues may also involve circulation, capture rolling, adhesion, crawling, transendothelial migration and interstitial migration (Chavakis et al., 2008; Emmanouil Chavakis, 2011; Nitzsche et al., 2017). Therefore, it is of great importance to enhance the migration ability of MSCs in order to improve cell engraftment and transplant efficiency. Up to now, studies about the migration and homing of exogenous MSCs for ICH treatment have been extremely rare.

Recently, it has been reported that the chemokines play pivotal roles in MSCs homing process, and MSCs exhibit chemotactic movement toward different chemokines (Stamatovic et al., 2003; Zhu et al., 2009; Guo et al., 2013; Li et al., 2015; Zhang et al., 2015; De Becker and Riet, 2016; Xiao et al., 2016; Xu et al., 2016). Fractalkine, or FKN for short, also known as CX3CL1, is one of the most abundant chemokines constitutively expressed on both neurons and astrocytes in the central nervous system (CNS) (Re and Przedborski, 2006; Miller et al., 2008). The specific receptor of Fractalkine/FKN, CX3CR1, is primarily expressed on microglial cells (Harrison et al., 1998). Meanwhile, an increased expression of fractalkine has been associated with development of ischemic stroke, brain and spinal cord injury and epilepsy (Xu et al., 2012; Poniatowski et al., 2017; Chen et al., 2018). Notably, elevated fractalkine levels at the lesion site can attract cells expressing CX3CR1, resulting in cell migration. Therefore, this study aimed to investigate whether overexpression of CX3CR1 in ADSCs can stimulate the cell migration to the injured brain, protect against neuronal apoptosis and improve functional recovery following experimental ICH.

## Materials and Methods

### Animals

Adult male C57BL/6 mice (8–10 weeks, 20–25 g, *n* = 95) and female Sprague-Dawley rats (5 weeks, 100–120 g, *n* = 75) were obtained from the Animal Center of Tongji Hospital of Tongji Medical College of Huazhong University of Science and Technology (Wuhan, China). The experimental procedures were approved by the Experimental Animal Ethics Committee of Huazhong University of Science and Technology.

### ICH Induction

Intracerebral hemorrhage was induced by a stereotactic intrastriatal injection of collagenase type VII (Sigma) as previously described (Pan et al., 2018). In brief, C57BL/6 male mice, 8–10 weeks old and weighing 20–25 g, were used in the experiments. Once anesthetized, the animals were fixed in a prone position in a stereotaxic frame, and a burr hole was drilled into the skull. Then, 0.1 U collagenase dissolved in 1 μl saline was stereotactically injected into the right basal ganglia (coordinates 0.2 mm anterior, 2.0 mm lateral, and 3.5 mm ventral to the bregma) at a rate of 0.2 μL/min. After injection, the syringe was left in place for 7 min and the needle was then withdrawn at a rate of 1 mm/min. After the suturing of wounds, all animals were monitored with free access to food and water.

### Assessment of ICH Model

After 8 h of modeling, the neurologic function was evaluated with Zea Longa’s five-grade scale (Longa et al., 1989). The five-point scoring criteria are as follows: a score of 0 indicated no neurologic deficit, a score of 1 (failure to extend left forepaw fully) indicated a mild focal neurologic deficit, a score of 2 (circling to the left) indicated a moderate focal neurologic deficit, and scores of 3 (falling to the left) and 4 (no spontaneous walk or a depressed level of consciousness) indicated severe focal deficits.

### Isolation and Characterization of ADSCs

Adipose-derived stem cells were isolated from the subcutaneous inguinal adipose tissues of female Sprague-Dawley rats (weighing 100–120 g) as previously described (Zhang et al., 2014). Briefly, fatty plaques were cut into pieces (1 mm^3^) and then digested with 0.1% collagenase Type I (Invitrogen) for 60 min at 37°C with gentle agitation. After centrifugation, the cell pellet was resuspended in the basal growth medium consisting of Dulbecco’s modified Eagle medium/Ham’s F12 (DMEM/F12; Hyclone) supplemented with 1% penicillin/streptomycin (Gibco), 1% L-glutamine (Gibco), and 10% fetal bovine serum (FBS; Sera Pro). Later, the suspension solution was filtered through a 40 μm cell strainer (BD Falcon) to remove debris. The remaining cells were seeded onto a 25 cm^2^ tissue culture bottle (Eppendorf) and incubated at 37°C in a humidified atmosphere of 5% CO_2_. After 1 h incubation, non-adherent cells were collected and transferred into a new culture bottle, in order to eliminate any impurities. The culture medium was changed in the following day and every 2–3 days thereafter. When reaching 80–90% confluency, the cells were detached by using a trypsin-EDTA solution (0.25% trypsin, 0.02% EDTA; Gibco) and were then passaged at a split ratio of 1:2. ADSCs between passages 2 and 5 were used for subsequent experiments.

Adipose-derived stem cells were analyzed for surface marker expression by flow cytometry. The isolated cells were incubated with fluorescein isothiocyanate (FITC)-conjugated antibodies against CD29, CD45 (eBioscience), phycoerythrin (PE)-conjugated antibodies against CD90, CD31 (BD Biosciences) and PBS (negative control) in a dark chamber at 4°C for 45 min. For each sample, at least 5 × 10^5^ cells were acquired for detection and analysis.

### Cell Transfection

The lentivirus for overexpressing CX3CR1 (LV-CX3CR1) and controls (Ubi-MCS-3FLAG-CBh-gcGFP-IRES-puromycin) were obtained commercially from GeneChem Corporation (Shanghai, China). When the ADSCs reached 30% confluency, lentiviral vectors were transferred to ADSC monolayer and incubated for 8 h [Multiplicity of Infection (MOI) = 40]. After lentivirus infection and transduction of ADSCs, CX3CR1-overexpressing cells (CX3CR1-GFP-ADSCs) and control cells (Ctr-GFP-ADSCs) were selected with 4 μg/ml puromycin. The effects of gene interference on CX3CR1 expression were verified using quantitative reverse transcription-polymerase chain reaction (qRT-PCR) and immunocytochemistry.

### Enzyme-Linked Immune Sorbent Assay (ELISA)

Animals were terminally anesthetized at indicated time points after collagenase-induced ICH. The ipsilateral perihematomal brain tissues (bregma: -2 to 2 mm) were then removed and homogenized in PBS with protease inhibitors (900 μl of PBS were added to each 100 mg brain tissues). Quantitative analysis of mouse fractalkine/FKN was performed using a double-antibody sandwich ELISA Array Kit (Elabscience, China) according to manufacturer’s instructions. In brief, tissue homogenates or standards were added to micro ELISA plate wells pre-coated with mouse fractalkine antibodies. Then, a biotinylated detection antibody specific for mouse fractalkine and avidin-horseradish peroxidase (HRP) conjugate was added into each micro plate well and incubated to wash away the free components. Afterward, the substrate solution was added into each well to yield a blue color, as a result of enzyme-substrate reaction. Immediately, sulfuric acid solution was added to terminate the reaction, which changed the color to yellow. The optical density (OD) was measured spectrophotometrically at a wavelength of 450 nm ± 2 nm. All determinations were performed in triple for each sample. Standard curve was generated to calculate the concentrations of fractalkine/FKN.

### Transwell Migration Assay

The migration assay was performed by using transwell plates with pore size of 8 μm (Corning Costar). The upper chambers were loaded with 2.3∼4 × 10^4^ ADSCs in 200 μl of DMEM/F12, while the lower chambers were loaded with 600 μl of DMEM/F12 (negative control), DMEM/F12 containing 10% FBS (positive control), and different concentrations of recombined fractalkine/ FKN dissolved in DMEM/F12 (Sigma). These chambers were incubated at 37°C with 5% CO_2_. After 24 h of incubation, non-migrated cells on the upper surface of the filter were removed by gentle scraping and washed with PBS. Next, the adherent cells were fixed in 4% paraformaldehyde (PFA) for 20 min and then stained with 1% crystal violet for 30 min. The average number of migrated cells was determined by examining five random fields per well. All experiments were performed in triplicate for each group.

### Cell Transplantation

Approximately 2∼4 × 10^5^ ADSCs in 2 μl PBS were transplanted into the right cerebral cortex (coordinates 0.1 mm posterior, 3.0 mm lateral, and 2.0 mm ventral to the bregma) of mice on the 3rd day after ICH. Mice were randomized allocated into three experimental groups: (1) PBS treatment; (2) Ctr-GFP-ADSC transplantation; and (3) CX3CR1-GFP-ADSC transplantation (*n* = 7 mice per group).

### Adhesive Removal Test

The adhesive removal test is a sensitive method that assesses sensorimotor deficits in mice (Bouët et al., 2007). This test was carried out blind at days 1, 3, 7, 14, and 21 after cell transplantation. The mice were trained for 5 consecutive days before surgery, to obtain an optimal level of performance and to reduce the inter-individual variations. Before the experiment, each mouse was placed into a transparent perspex box (15 cm × 25 cm) for a habituation period of 60 s. Thereafter, two small pieces of adhesive surgical tape of equal size (0.3 cm × 0.4 cm) were placed with an equal pressure on each forepaw, covering their hairless parts (i.e., the three pads, thenar, and hypothenar). The order of placement (right or left) was alternated between each animal and session. The mice then transferred back to the perspex box, and the time for sensing and removing each adhesive tape were recorded with a maximum of 180 s.

### Cylinder Test

The cylinder test was used to assess forelimb preference and asymmetry in each mouse on days 1, 3, 7, 14, and 21 after cell transplantation (Vahid-Ansari et al., 2016). Mice were placed in a transparent cylinder (diameter: 10 cm, height: 16.5 cm) for 10 min and the number of forepaw contacts to the cylinder wall was counted. The final score was calculated as: (Right forelimb movement – Left forelimb movement)/(Right forelimb movement + Left forelimb movement + both movement). The behavioral tests were performed by the two co-authors blind to the group of mice.

### TTC Staining

Animals were sacrificed 3 days following transplantation of ADSCs. Hematoma volume was quantified using TTC staining method. Mice were perfused with normal saline solution, and then the brains were dissected and sectioned into 2 mm coronal sections from the frontal pole. The slices were incubated in a 2% solution of TTC at 37°C for 30 min. The sections were subsequently fixed with 4% paraformaldehyde solution. The volume of hematoma was calculated by summing the relative areas of hematoma from the individual sections and multiplying them by the distance between the sections.

### Immunocytochemistry and Immunofluorescence Staining

Mice were transcardially perfused with saline and 4% PFA. The brains were dissected out and post-fixed in 4% PFA for 24 h, followed by dehydration with 25 and 30% sucrose. The brain tissues were cut into 30 μm thick sections (Leica CM1950, Germany) and picked up on a glass slide for staining. The tissue sections or adherent cells were then fixed in 4% PFA, washed three times with PBS, and incubated in 0.5% Triton X-100 for 30 min. After blocking with 5% Bovine Serum Albumin (BSA) for 1 h at room temperature, slices or cells were incubated with primary antibodies rabbit anti-CX3CL1 (1:500, Abcam, Cambridge), mouse anti-NeuN (1:500, Abcam, Cambridge), mouse anti-GFAP (1:500, Santa Cruz, CA, United States), rabbit anti-CX3CR1 (1:100, Abcam, Cambridge), goat anti-Iba-1 (1:100, Abcam, Cambridge), or chicken anti-GFP (1:1000, Abcam, Cambridge) at 4°C overnight. Secondary antibodies of FITC conjugated goat anti rabbit IgG (1:100, Boster Biological Technology Co., Ltd., United States), Cy3 conjugated goat anti mouse IgG (1:100, Boster Biological Technology Co., Ltd., United States), Cy3 conjugated donkey anti mouse IgG (1:200, Servicebio, China), Alexa Fluor^®^ 594 donkey anti-goat IgG (1:400, life technologies, the United States) or Alexa Fluor^®^ 488 conjugated Goat anti-chicken IgG (1:500, Abcam, Cambridge) were subsequently incubated for 1 h at 37°C. Counterstaining was performed with 4′,6-diamidino-2-phenylindole (DAPI; Beyotime, China).

### Terminal Deoxynucleotidyltransferase-Mediated dUTP End Labeling (TUNEL) Staining

The paraffin-embedded tissue sections were prepared for TUNEL staining with the *in situ* Cell Death Detection Kit, POD (Roche, Switzerland). The numbers of TUNEL-positive cells were quantified with the use of Image-Pro Plus software. To determine the frequencies of apoptotic cells, five microscopic fields were examined for each sample. The apoptotic index was defined as the average proportion of TUNEL-positive cells in each area.

### Western Blot

The total protein concentration in cells and tissues was determined using bicinchoninic acid assay (BCA) (Beyotime, China) according to manufacturer’s instructions. After sodium dodecyl sulfate polyacrylamide gel electrophoresis (SDS-PAGE), the detected proteins were transferred onto nitrocellulose filter (NC) membranes. Subsequently, the membranes were blocked in 5% skim milk for 1 h at room temperature with gentle shaking. After that, the membranes were incubated with primary antibodies of rabbit anti-CX3CR1 (1:500, Abcam, Cambridge), mouse anti-bcl-2 (1:500, Santa Cruz, CA, United States), mouse anti-caspase-3 (1:500, Santa Cruz, CA, United States), and/or mouse anti-β-actin antibody (1:1000, Proteintech, China) at 4°C overnight, followed by 1 h incubation with anti-rabbit IgG (H+L) (DyLight 800 Conjugate; Cell Signaling Technology, United States) or anti-mouse IgG (H+L) (DyLight 800 Conjugate; Cell Signaling Technology, United States) secondary antibody at room temperature. The protein bands were visualized with Odyssey Infrared Imaging System (LI-COR Biosciences, Lincoln, NE, United States), and the intensity of each band was analyzed using Image J (National Institutes of Health, United States).

### Statistical Analysis

All experimental data from three independent experiments were analyzed using SPSS software (version 20.0 for Windows, SPSS Inc., United States) and the results were expressed as mean ± standard deviation (SD). One-way analysis of variance (ANOVA) followed by Tukey’s *post hoc* test was used for parametric data and multiple comparisons between groups. In Figure 5D, two-way ANOVA was used to assess the main effect of each independent variable and the latent interaction between them. *P*-values of less than 0.05 were considered statistically significant.

## Results

### Increased Expression of Fractalkine/FKN After ICH

**FIGURE 1:**
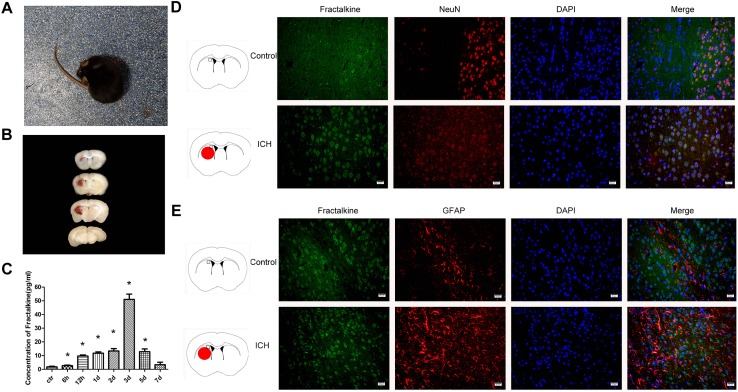
Mice began to turn around spontaneously 8 h after ICH **(A)**, and hematoma was seen in the right basal ganglia at day 3 **(B)**. ELISA showed that the expression level of fractalkine could be increased at early time points, with a peak at day 3 after ICH **(C)** (*n* = 3 each group, ^∗^*P* < 0.001, as compared to normal control group). Fractalkine, mainly produced by neurons and astrocytes, was constitutely expressed in basal ganglion of the brain tissue (**D,E**, scale bar = 20 μm; the square in the schema diagram showed the area where from the pictures were taken).

After the establishment of ICH model, the mice exhibited weakness of left limbs, uncoordinated movement and spontaneous rotation (Figure 1A). A proper size of hematoma could be induced with 0.1 U collagenase (Figure 1B). Mice scoring grades 1–3 of Zea Longa’s scale were selected for further experiments. ELISA method was used to detect the dynamic changes of fractalkine in the brain tissue homogenates at 6 h, 12 h, and days 1, 2, 3, 5, and 7 after ICH. The results indicated that the expression levels of fractalkine were significantly increased at early time points, with a peak at day 3 after ICH (Figure 1C). Therefore, we transplanted ADSCs on the 3rd day after ICH in the following *in vivo* experiments. The immunofluorescence staining revealed that fractalkine was constitutively expressed in normal brain tissues by neurons and astrocytes, distributed mainly in the cortex, basal ganglia and other regions (Figure 1D,E).

### Characterization of ADSCs

**FIGURE 2:**
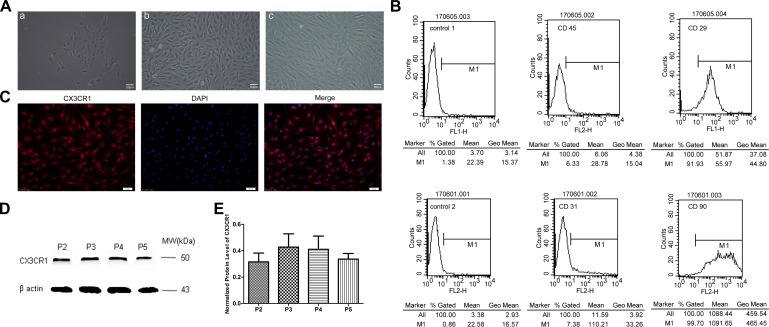
Cellular morphology of ADSCs **(A)**. The primary ADSCs displayed short bar shapes initially (**a**, the 3rd day of cell culture), and maintained spindle-shaped morphology thereafter (**b**, the 7th day of cell culture) and could stable proliferate (**c**, passage 3 ADSCs) (scale bar = 50 μm). P2 ADSCs were positive for CD29 (91.93%) and CD90 (99.70%), and the expression level of CD45 (6.33%) and CD31 (7.38%) were very low **(B)**. P2-P5 ADSCs could stably expressed CX3CR1 in both membrane and cytoplasm detected by immunocytochemistry (**C**, scale bar = 50 μm) and western blot **(D,E)**.

Primary ADSCs exhibited short bar shapes during the early stages, and maintained a spindle-shaped morphology thereafter (Figure 2A). Flow cytometry analysis showed that these cells were positive for MSC markers CD29 (91.93%) and CD90 (99.70%), while the expression of hematopoietic cell markers CD45 (6.33%) and CD31 (7.38%) were relatively low (Figure 2B).

### CX3CR1 Protein Expression in ADSCs

The results of immunocytochemical staining revealed that ADSCs were found to express CX3CR1, which distributed mainly in the cell membrane and cytoplasm (Figure 2C). Western blot results demonstrated that the stable expression of CX3CR1 could be detected in ADSCs at passage 2–5 (Figure 2D,E).

### Establishment of Stable CX3CR1 Overexpressing ADSCs

**FIGURE 3:**
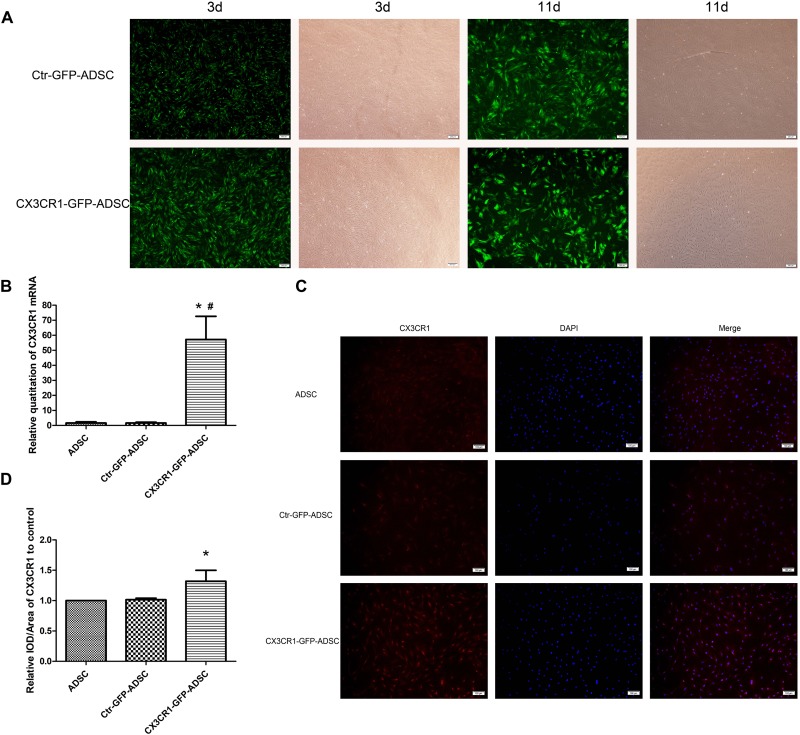
After transfection with lentivirus and screen with puromycin, most ADSCs expressed the tag protein of GFP on the 3rd day, and the transfection rate could still remain at 80–90% till the 11th day (**A**, scale bar = 200 μm). qRT-PCR showed that mRNA expression levels of CX3CR1 in CX3CR1-GFP-ADSCs were significantly higher than the other two groups (**B**, ^∗^*P* < 0.01, as compared with ADSC group; ^#^*P* < 0.01, as compared with Ctr-GFP-ADSC group). IOD analysis of immunocytochemical images showed a higher CX3CR1 protein expression level in CX3CR1-GFP-ADSCs (**C,D**, scale bar = 100 μm; ^∗^*P* < 0.05, as compared with ADSC group, IOD, integrated optical density).

Following lentivirus transfection and puromycin screening, the transfection rate of ADSCs could remain up to 80–90% until day 11 of transfection (Figure 3A). The mRNA and protein expression levels of CX3CR1 were significantly higher (*P* < 0.05) in CX3CR1-GFP-ADSCs than normal ADSCs (Figure 3B–D).

### Overexpression of CX3CR1 Augments ADSC Migration *in vitro*

**FIGURE 4:**
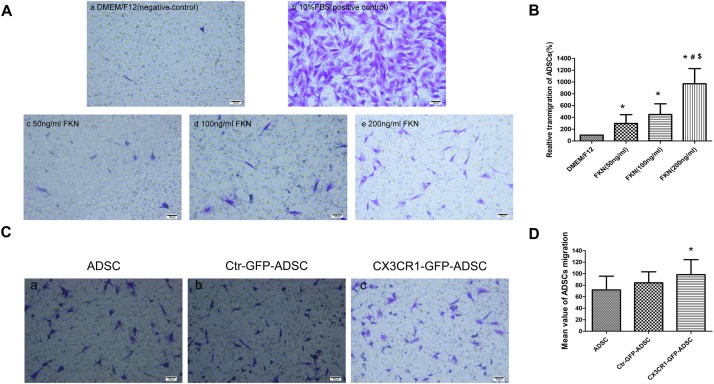
*In vitro* transwell migration assay. Chemotactic movements of ADSCs toward fractalkine/FKN *in vitro* **(A)**. The lower compartment was filled with 600 μl DMEM/F12 (negative control) **(a)**, 10% FBS (positive control) **(b)**, 50 ng/ml FKN **(c)**, 100 ng/ml FKN **(d)**, and 200 ng/ml FKN **(e)** in DMEM/F12. The upper chamber were cell suspensions of ADSCs (2.3–2.4 × 10^4^ cells/200 μl). It was found that the number of ADSCs directed migration to the bottom of chamber increased significantly with the increase of the concentration of fractalkine/FKN (**B**, relative transmigration of ADSCs = migration number of experimental group/migration number of negative control group,^∗^*P* < 0.01, as compared with DMEM/F12 group; ^#^*P* < 0.05 as compared with FKN (50 ng/ml) group; ^$^*P* < 0.01, as compared with FKN (100 ng/ml) group. *In vitro* migration ability of ADSCs after overexpressing CX3CR1 **(C)**. There were totally three groups: Ctr-GFP-ADSCs, CX3CR1-GFP-ADSCs, and untransfected ADSCs. The corresponding ADSCs (4 × 10^4^ cells/200 μl) were placed in the upper chamber, and the lower chamber was loaded with 200 ng/ml FKN in the DMEM/F12 solution. The results showed that compared with untransfected ADSCs, CX3CR1 – GFP – ADSCs migrated more toward FKN (**D**, ^∗^*P* < 0.01, as compared with ADSC group).

Transwell migration assay was used to detect the chemotactic movements of ADSCs toward fractalkine/FKN. The results showed that the number of ADSCs migrated to the bottom of chamber was significantly increased with the elevated concentrations of fractalkine/FKN (Figure 4A,B). So FKN with the concentration of 200 ng/ml was loaded in the lower chamber in the following transwell migration assays to detect the migration ability of CX3CR1-GFP-ADSCs. The upper chamber was filled with (1) untransfected ADSCs, (2) Ctr-GFP-ADSCs, and (3) Ctr-GFP-ADSCs, and then the number of migrated cells toward FKN was compared. Finally, the augmented *in vitro* migration ability of CX3CR1-GFP-ADSCs was confirmed, as it migrated more efficiently toward FKN compared to untransfected ADSCs (*P* < 0.05). However, there were no statistically significant differences in the migration rate between Ctr-GFP-ADSCs and untransfected ADSCs (Figure 4C,D).

### Overexpression of CX3CR1 Augments ADSCs Homing to the Hemorrhagic Brain

**FIGURE 5:**
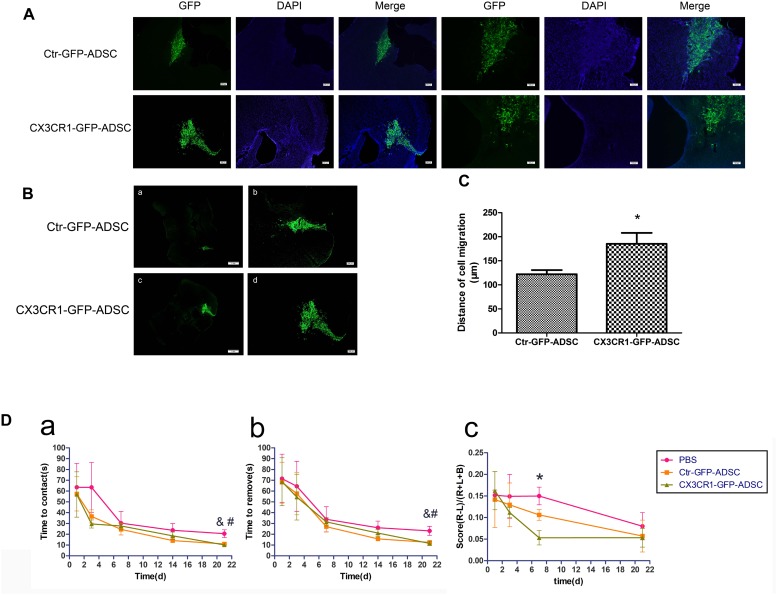
Three days after transplantation, most of cells were distributed near the transplantation site (**A**, scale bar = 200 μm, 100 μm). Compared with Ctr-GFP-ADSC group, more CX3CR1 – GFP – ADSCs migrated toward the lesion loci (**B**, scale bar = 1 mm, 200 μm), and the distance of migration was comparatively longer (**C**, ^∗^*P* < 0.05, as compared with Ctr-GFP-ADSC group). Adhesive removal test and cylinder test was use to assess functional recovery after cell transplantation in ICH **(D)**. Mice in both cell transplantation groups had a tendency to sense and remove stickers faster in the affected extremity on the 21st day after transplantation (**a**, time to contact the stickers in the left forepaw; **b**, time to remove the stickers in the left forepaw, *n* = 7 each group, ^&^*P* < 0.1, Ctr-GFP-ADSC group vs. PBS group; ^#^*P* < 0.1, CX3CR1-GFP-ADSC group vs. PBS group). In the cylinder test, scores were lower in CX3CR1-GFP-ADSC group than PBS group on days 7 post-transplantation, but no difference was observed between the Ctr-GFP-ADSC and PBS group (**c**, the final scores to assess forelimb preference and asymmetry, *n* = 7 each group, ^∗^*P* < 0.05, CX3CR1-GFP-ADSC group vs. PBS group).

Most of the transplanted cells were distributed throughout the transplant area and survived on the 3rd day after transplantation (Figure 5A). Few ADSCs migrated to the lesion site over comparatively short distances. In contrast, more cells were seen to migrate to the target site in the CX3CR1-GFP-ADSC group as compared to the Ctr-GFP-ADSC group, and the distance of migration was comparatively longer (Figure 5B,C). We also detected the immunological response after transplantation. It was found that there was no obvious microglial activation against rat-derived ADSCs (Supplementary Figure S1).

### ADSCs Overexpressing CX3CR1 Promote Functional Recovery After ICH

At day 21 after cell transplantation, the mice in both Ctr-GFP-ADSC and CX3CR1-GFP-ADSC groups had a tendency to sense and remove stickers earlier and faster than those in the PBS group, as indicated by adhesive removal test (Figure 5Da,b and Table 1, 0.05<*P* < 0.1). In the cylinder test, scores were lower in CX3CR1-GFP-ADSC group than PBS group on days 7 post-transplantation, but no difference was observed between the Ctr-GFP-ADSC and PBS group (Figure 5Dc and Table 1, *P* < 0.05).

**Table 1:** The effects of ADSCs transplantation on functional recovery following intracerebral hemorrhage.

A. Adhesive removal test			

Variants	Group	Days after transplantation				
		
		Day 1	Day 3	Day 7	Day 14	Day 21
Time to contact in the left(s)	PBS	63.57 ± 22.03	63.43 ± 23.06	30.29 ± 10.87	23.71 ± 6.19	20.57 ± 3.58
	Ctr-GFP-ADSC	57.43 ± 15.85	36.57 ± 6.08	24.57 ± 5.20	14.14 ± 1.44	11.00 ± 0.98*
	CX3CR1-GFP-ADSC	56.86 ± 20.95	29.57 ± 3.76	27.71 ± 3.15	18.57 ± 3.54	10.14 ± 1.86*
Time to remove in the left(s)	PBS	71.57 ± 22.60	64.43 ± 23.23	33.86 ± 11.66	26.00 ± 6.24	23.14 ± 4.20
	Ctr-GFP-ADSC	68.14 ± 18.54	57.71 ± 19.69	27.00 ± 5.00	15.71 ± 1.92	12.43 ± 1.11*
	CX3CR1-GFP-ADSC	68.71 ± 22.26	54.43 ± 21.22	31.43 ± 3.77	21.14 ± 3.41	11.57 ± 2.00*
Time to contact in the right(s)	PBS	20.86 ± 2.90	14.71 ± 3.08	11.57 ± 2.03	9.29 ± 0.64	6.29 ± 0.57
	Ctr-GFP-ADSC	19.14 ± 2.13	14.57 ± 3.30	11.86 ± 1.87	9.14 ± 1.99	7.29 ± 1.15
	CX3CR1-GFP-ADSC	19.00 ± 2.73	13.86 ± 2.51	11.14 ± 1.53	10.43 ± 2.18	7.29 ± 1.15
Time to remove in the right(s)	PBS	23.43 ± 3.21	20.43 ± 2.79	13.57 ± 2.17	11.14 ± 0.70	7.71 ± 0.97
	Ctr-GFP-ADSC	22.00 ± 2.73	18.43 ± 3.26	14.71 ± 2.99	10.71 ± 2.17	8.29 ± 1.34
	CX3CR1-GFP-ADSC	22.86 ± 4.75	18.00 ± 2.40	14.86 ± 3.38	12.14 ± 2.31	9.00 ± 1.62

**B. Cylinder test**						

**Variants**	**Group**		**Days after transplantation**			
			
			**Day 1**	**Day 3**	**Day 7**	**Day 21**

Forelimb preference and asymmetry final scores	PBS		0.15 ± 0.01	0.15 ± 0.05	0.15 ± 0.02	0.08 ± 0.03
	Ctr-GFP-ADSC		0.14 ± 0.06	0.13 ± 0.05	0.11 ± 0.01	0.06 ± 0.04
	CX3CR1-GFP-ADSC		0.16 ± 0.04	0.11 ± 0.01	0.05 ± 0.02^#^	0.05 ± 0.02


*^∗^*P* < 0.1 compared to PBS group; ^#^*P* < 0.05 compared to PBS group.*

### ADSCs Overexpressing CX3CR1 Reduce ICH-Induced Cell Death

**FIGURE 6:**
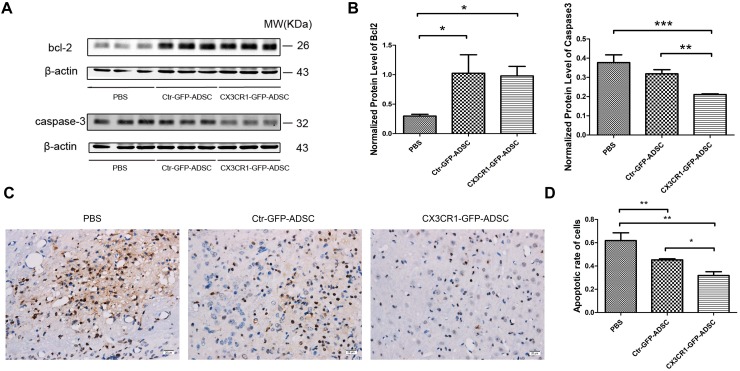
Western blot showed that both cell transplantation group could reduce the expression of bcl-2, while the CX3CR1-GFP-ADSC group could more significantly lower the expression of caspase-3 compared to Ctr-GFP-ADSC and PBS group (**A,B**, ^∗^*P* < 0.05; ^∗∗^*P* < 0.01; ^∗∗∗^*P* < 0.001). TUNEL staining showed that both cell transplantation groups could reduce the apoptosis rate of the foci (**C**, scale bar = 20 μm), and a lower apoptosis rate was found in the CX3CR1-GFP-ADSC group (**D**, ^∗^*P* < 0.05; ^∗∗^*P* < 0.01).

The results of Western blot analysis revealed that anti-apoptotic protein bcl-2 expression level was significantly higher (*P* < 0.05) in the cell transplantation group (i.e., Ctr-GFP-ADSC and CX3CR1-GFP-ADSC groups) than that in the PBS group. However, no significant difference was observed between the two cell transplantation groups (Figure 6A,B). Meanwhile, the expression level of pro-apoptotic protein caspase-3 was significantly decreased (*P* < 0.05) in the CX3CR1-GFP-ADSC group compared to Ctr-GFP-ADSC and PBS groups (Figure 6A,B). TUNEL assay showed significantly reduced number of apoptotic cells in the cell transplantation groups compared to the PBS group (*P* < 0.01). Similarly, a significant difference (*P* < 0.05) was observed between Ctr-GFP-ADSC group and CX3CR1-GFP-ADSC group with respect to the apoptosis rate (Figure 6C,D). In addition, we also detected the hematoma volume in different groups. TTC staining showed that reduced volumes of hematoma were seen in both cell transplantation groups, but no difference was observed between the Ctr-GFP-ADSC and CX3CR1-GFP-ADSC group (Supplementary Figure S2).

## Discussion

Fractalkine/FKN, or CX3CL1, is the only member of CX3C subfamily of chemokines with two isoforms (Poniatowski et al., 2017). The membrane-bounded form of fractalkine is related to the adhesive effects, while the soluble form enhances the recruitment and activation of inflammatory cells (Réaux-Le Goazigo et al., 2013). Unlike most chemokine peptides, fractalkine can be expressed in non-hematopoietic tissues, including brain, kidney, lung, and heart. In particular, fractalkine is constitutively expressed in the CNS at relatively high levels, which produced mainly by neurons and astrocytes (Harrison et al., 1998; Re and Przedborski, 2006; Sciumè et al., 2010; Réaux-Le Goazigo et al., 2013). Meanwhile, its unique receptor CX3CR1, is primarily expressed on microglia (Donohue et al., 2012; Réaux-Le Goazigo et al., 2013). Previous studies reported that cerebrospinal fluid levels of soluble fractalkine are elevated in patients with traumatic brain injury and inflammatory brain diseases (Kastenbauer et al., 2003; Rancan et al., 2004). Similarly, increased serum levels of fractalkine have been reported in multiple sclerosis and systemic lupus erythematosus with neuropsychiatric involvement (Kastenbauer et al., 2003; Yajima et al., 2005). Here, we firstly detect the dynamic changes of fractalkine in brain tissue homogenates after ICH. We found that the levels of fractalkine can be increased at early time points, with a peak on the 3rd day after ICH. Despite controversies over the neuroprotective and neurotoxic roles of fractalkine (Donohue et al., 2012; Réaux-Le Goazigo et al., 2013), it is of great significance to study the dynamic changes of fractalkine expression after CNS diseases or injury. It’s known that elevated fractalkine can attract endogenous or exogenous CX3CR1-expressing cells to migrate toward lesion loci. Consequently, studying the dynamic change of fractalkine can help to decide the time point of cell transplantation so that the grafting efficiency of stem cell therapy might be further improved through modifying infused cells or internal microenvironment. In addition, fractalkine expression has been considered as an essential biomarker in predicting the severity and prognosis of diseases (Kim et al., 2008; Donohue et al., 2012; Grosse et al., 2014).

Stem cell therapy is a promising therapeutic strategy for neurological diseases. It is generally believed that an adequate number of stem cells homing and migrating toward lesion site is important for efficient cell transplantation (Chavakis et al., 2008; Karp and Leng, 2009). Although infused cells tend to migrate to the lesion site, the decreased rate of cell homing, retention and survival can be the major limitations (Karp and Leng, 2009; Emmanouil Chavakis, 2011; Tang et al., 2015). MSC homing has been first defined by Karp and Leng (2009), as the arrest of MSCs within the vasculature of a tissue followed by transmigration across the endothelium. Recently, Nitzsche et al. (2017) expanded the definition of MSC homing to non-systemic and systemic homing according to MSC administration routes. However, the exact mechanisms underlying the homing and migration of MSCs are not fully understood. It is proposed that systemic homing require a coordinated multistep process involving ingress to the circulation, extravasation at the lesion vicinity, and interstitial migration toward the target site. For non-systemic homing, locally infused MSCs directly possess interstitial migration following activation and polarization (Chavakis et al., 2008; Emmanouil Chavakis, 2011; Nitzsche et al., 2017). Chemokines play an important role during the process of cell migration. In this study, we found that the expression level of fractalkine increased after ICH, and different passages of ADSCs express its receptor CX3CR1. Besides, the chemotactic movements of ADSCs toward fractalkine have been confirmed *in vitro*. So, we want to detect whether ADSCs overexpressing CX3CR1 could migrate more to the hemorrhagic site to achieve a higher therapeutic efficiency with the same former transplantation dosage.

Stem cell transplantation involves various delivery routes. Local infusion can be used to inject MSCs directly into the tissues of interest, but this strategy may not be clinically feasible due to its high invasiveness and diffusion limitations of nutrients and oxygen (Karp and Leng, 2009). Intravenous delivery has the advantages of being more convenient, less invasive and much safer process than intrathecal or intracerebral administration (Lanza et al., 2009), but the MSCs homing is limited. It’s reported that a high engraftment rate might be achieved by intra-arterial delivery, through the bypassing of filtration organs (Walczak et al., 2008). It still seems unclear whether systemically infused MSCs can actively migrate across BBB. MSCs were found to transmigrate across the brain microvascular endothelial cells monolayers *in vitro* through the transient intercellular gaps between them, but the endothelial cell monolayers do not fully recapitulate the *in vivo* BBB properties (Matsushita et al., 2011). In this study, we applied intracortical transplantation method to preliminarily investigate the short-term distribution and migration ability of CX3CR1 overexpressing ADSCs. Since the site of transplantation is relatively superficial, it is conducive to oxygen and nutrition diffusion. In addition, the short distance from the injection site to the injury center is favorable for observing the migration of cells.

The stable overexpression of CX3CR1 ADSCs (CX3CR1-GFP-ADSCs) were established with lentivirus transfection. Our findings demonstrated that the *in vitro* migration ability of ADSCs was increased after overexpressing CX3CR1. Additionally, the infused cells could survive until day 3 after transplantation, and a small part of ADSCs could migrate to the hemorrhagic brain site. In this study, CX3CR1-GFP-ADSC group showed better efficiency as compared to Ctr-GFP-ADSC group, with respect to the number of cells migrate toward the target region and their migration distances. There are various strategies to improve the migration ability of stem cells up to now (Li et al., 2017). And genetic manipulation remains one of the stable and efficient methods to improve their migration and mobility. However, the risk of malignant transformation and time-consuming should be noticed during the process (Liu et al., 2014). The ultimate aim of this study is to achieve increased homing and engraftment of MSCs by using lower or the same dosage of transplanted cells, in order to decrease the costs and duration of cell preparation (Maijenburg et al., 2012; Li et al., 2017). Interestingly, we observed the positive effects of ADSCs overexpressing CX3CR1 in reducing neuronal apoptosis and improving functional recovery after ICH. A tendency of improved sensory and motor functions and reduced expression of caspase-3 and increased expression of bcl-2 in CX3CR1-GFP-ADSC transplantation group were found in our research. Furthermore, a lower apoptosis rate around the foci was observed in CX3CR1-GFP-ADSC group as compared to Ctr-GFP-ADSC group. However, there are some limitations for our study. We transplanted rat ADSCs to the mice in this experiment, and human ADSCs will be used in our later studies, which is closer to the actual clinical situation. In addition, we should observe for a longer time to determine whether the transplanted ADSCs have teratogenic effects.

## Conclusion

In summary, ADSCs overexpressing CX3CR1 demonstrated an increased migration ability both *in vitro* and *in vivo*, and may improve therapeutic efficiency in the ICH model.

## Ethics Statement

The study was carried out in accordance with the recommendations in the Guide for the Care and Use of Laboratory Animals of the National Institutes of Health. The protocol was approved by competent ethics committees at Huazhong University of Science and Technology.

## Author Contributions

GL and HY were responsible for the cellular and animal experiments. GL performed the ELISA, immunohistochemical staining, qRT-PCR, and cell transplantation experiments. HY was responsible for the animal model and preparation of frozen sections of brain tissues. GL and HD performed the Western blot experiments. GL and JW performed the behavioral tests. NL, PZ, YT, YH, YZ, CP, QL, and ZT were responsible for the conception or design of the work. QL and ZT drafted the work or revisited it critically. All authors approved the final version of the manuscript.

## Conflict of Interest Statement

The authors declare that the research was conducted in the absence of any commercial or financial relationships that could be construed as a potential conflict of interest.

## Supplementary Material

Figure S1 Microglial activation against rat-derived ADSCs around the injection site 3 days after transplantation (scale bar = 50 μm).

Figure S2 TTC staining showed that reduced volumes of hematoma were seen in both cell transplantation groups, but no difference was observed between the Ctr-GFP-ADSC and CX3CR1-GFP-ADSC group (**A,B**, *n* = 3 each group, ^∗^*P* < 0.0001).
