# Supplementary figures and images for: Overexpression of CX3CR1 in Adipose-Derived Stem Cells Promotes Cell Migration and Functional Recovery After Experimental Intracerebral Hemorrhage
Source: Front Neurosci. 2019 May 8;13:462. doi: 10.3389/fnins.2019.00462 (PMC6517499; doi:10.3389/fnins.2019.00462)

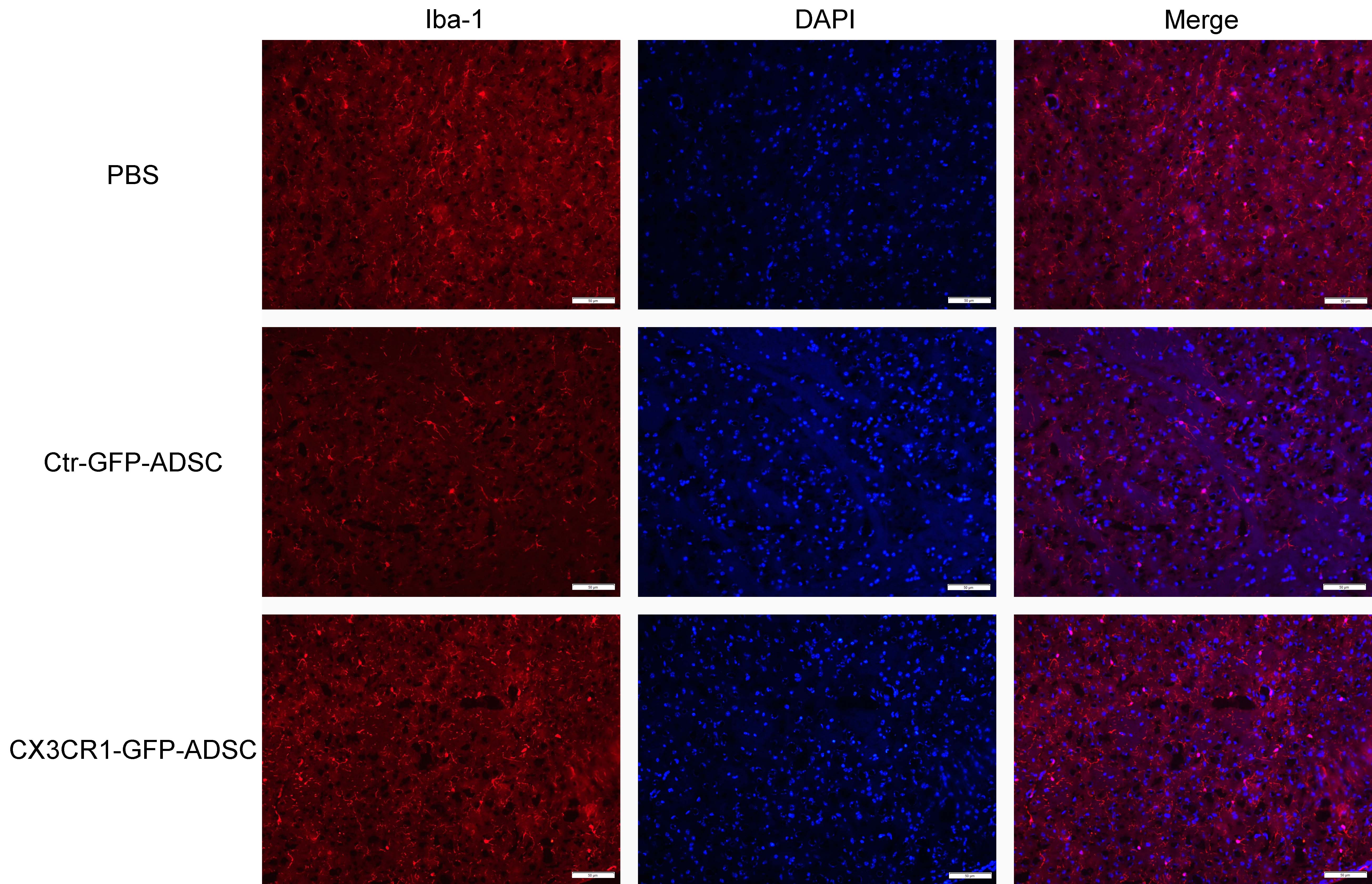

Supplement: Figure S1 — Microglial activation against rat-derived ADSCs around the injection site 3 days after transplantation (scale bar = 50 μm). [file Image_1.JPEG]

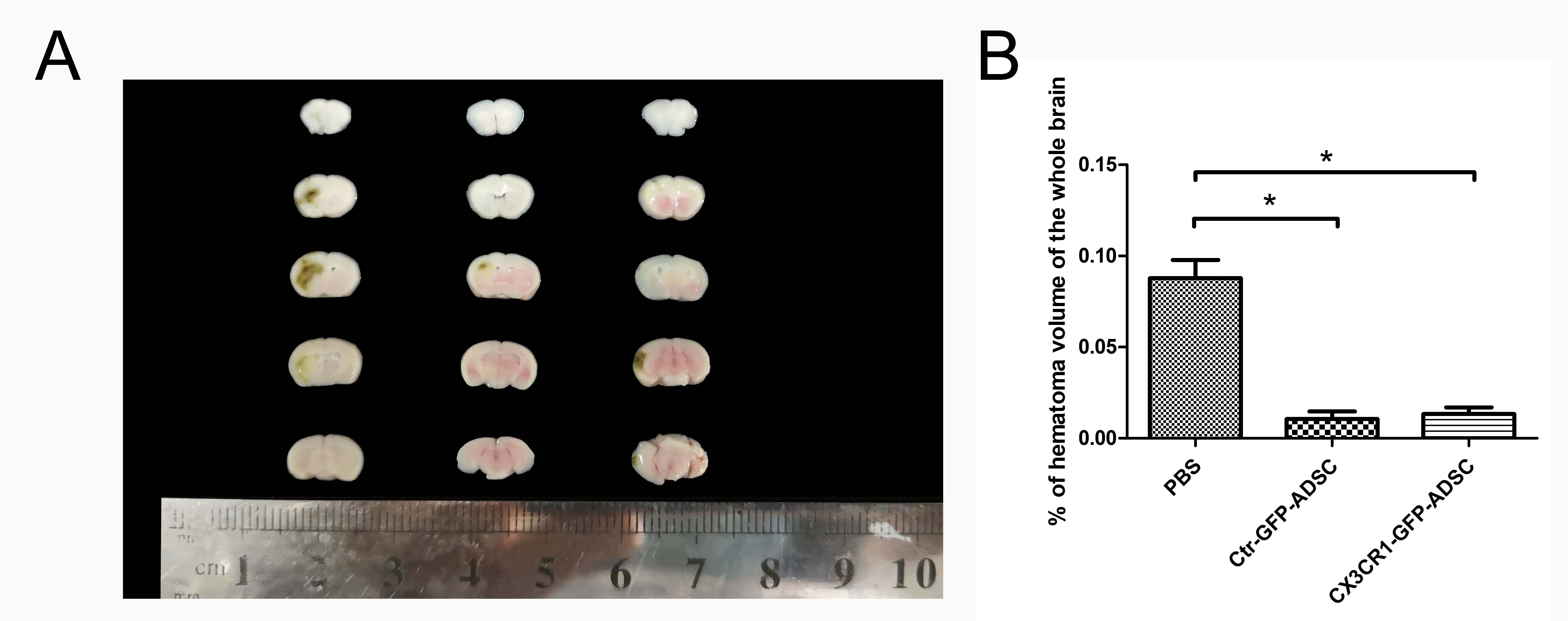

Supplement: Figure S2 — TTC staining showed that reduced volumes of hematoma were seen in both cell transplantation groups, but no difference was observed between the Ctr-GFP-ADSC and CX3CR1-GFP-ADSC group (A,B, n = 3 each group, ∗P < 0.0001). [file Image_2.JPEG]
